# Poxvirus Viability and Signatures in Historical Relics

**DOI:** 10.3201/eid2002.131098

**Published:** 2014-02

**Authors:** Andrea M. McCollum, Yu Li, Kimberly Wilkins, Kevin L. Karem, Whitni B. Davidson, Christopher D. Paddock, Mary G. Reynolds, Inger K. Damon

**Affiliations:** Centers for Disease Control and Prevention, Atlanta, Georgia, USA

**Keywords:** smallpox, smallpox virus, poxvirus, viruses, variola, orthopoxvirus, vaccinia, viability, signatures, artifacts, historical relics, mummies

## Abstract

Although it has been >30 years since the eradication of smallpox, the unearthing of well-preserved tissue material in which the virus may reside has called into question the viability of variola virus decades or centuries after its original occurrence. Experimental data to address the long-term stability and viability of the virus are limited. There are several instances of well-preserved corpses and tissues that have been examined for poxvirus viability and viral DNA. These historical specimens cause concern for potential exposures, and each situation should be approached cautiously and independently with the available information. Nevertheless, these specimens provide information on the history of a major disease and vaccination against it.

Chinese writings from 1122 bce contain references to smallpox-like disease, and it has been hypothesized that smallpox caused the death of Ramses V in Egypt in ≈1157 bce because poxvirus-like lesions were seen on the mummy (*1*,*2*). The most recent epidemics of smallpox occurred through the 1900s, and the last naturally occurring case of smallpox was seen in Somalia in 1977 (*3*). Historical tissue specimens and artifacts yield useful information about the history of and vaccination against smallpox. However, the absolute viability of poxviruses in well-preserved samples has not been determined. Thus, it is not known what risks these artifacts might pose to persons who come into contact with them.

Smallpox is caused by variola virus (genus *Orthopoxvirus*). Illness is characterized by 3 phases: incubation, prodrome, and rash. The incubation phase is ≈10–14 days. During the prodromal period, which lasts 2–4 days, persons with smallpox typically have fever, malaise, vomiting, headache, backache, and myalgia. The rash phase can be moderate or severe and is characterized by a centrifugal distribution of lesions in the same stage of development (Figure 1) in any 1 area of the body. Lesions, including their crusts, contain infectious virus through all stages of the rash. Thus, contact with infectious lesion exudate and tissue (including crusts) can result in virus transmission. However, the most common route of transmission is inhalation of infectious respiratory droplets. Patients who survive an infection often have life-long scarring, and they maintain some level of immunity to orthopoxvirus infection (*1*,*4*,*5*).

**Figure 1:**
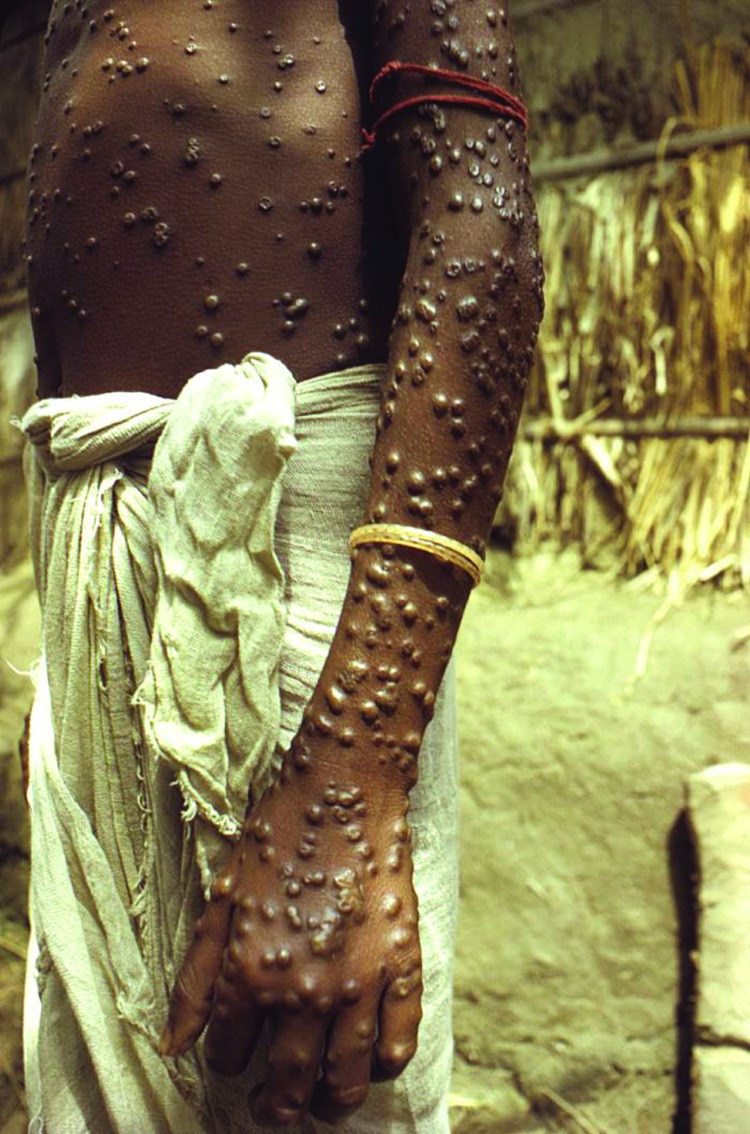
Patient with smallpox. Photograph by Jean Roy, provided by the Public Health Image Library, Centers for Disease Control and Prevention, Atlanta, GA, USA.

Elimination and eradication of smallpox were feasible, in part, because there is no animal reservoir for variola virus. The World Health Organization (WHO) announced worldwide eradication of smallpox in 1980. Successful eradication was accomplished by vaccinating populations and contacts of ill persons with live vaccinia virus, a closely related orthopoxvirus that confers immunity to variola virus. Once smallpox was eradicated, WHO recommended that routine vaccination be discontinued and that the vaccine be used only for select groups at risk for exposure to orthopoxviruses. Thus, persons born after 1980 are likely to not have residual immunity (*1*,*6*). Intentional arm-to-arm transfer of virus by dried scab or lesion exudate from a recent vaccinee was common in nineteenth century Great Britain (*7*). Crusts were collected, stored, and sent to others to aid vaccination before mass production and distribution of vaccine stocks. Scab material from patients with smallpox was often used for variolation, the practice of deliberately infecting a person with smallpox to (hopefully) induce a mild infection and subsequent immunity. Variolation continued into the twentieth century in some regions (*1*).

Present-day stocks of variola virus are maintained at 2 WHO reference laboratories: the Centers for Disease Control and Prevention (CDC) (Atlanta, GA, USA) and the State Research Center of Virology and Biotechnology (VECTOR) (Koltsovo, Russia). There is concern that if variola virus is present outside these 2 laboratories, its accidental or intentional release could cause illness in a population increasingly composed of unvaccinated persons. Anecdotal reports and formal scientific evidence have not ruled out the possibility that the virus may survive prolonged periods in preserved skin and tissue material, such as those that might be on display in museums, or in unearthed human remains. For example, permafrost is an environment that closely mirrors laboratory freezer storage of live virus, and the maintenance of viable smallpox virus in human remains found in such an environment has been debated (*8*). Environmental contamination with potentially live variola virus recovered from historical relics could threaten our confidence that the disease has been eradicated. In addition to immediate public health concern about such relics, there is much to be gained from investigation of artifacts in terms of scientific and historical interests.

We reviewed experimental data that address virus longevity in a variety of environments. There are several accounts of historical smallpox specimens in the form of unearthed remains and lesion crusts. The Poxvirus Laboratory at CDC recently reviewed this data and reexamined specimens from the inventory to revisit the existence of sections of intact DNA by using more modern methods. We also address the role of public health and scientific interest in such specimens.

We found published articles by searching PubMed for material on virus viability and historical specimens. Search terms included viability smallpox, viability variola, viability orthopoxvirus, and smallpox and corpse. References from articles that cited previous work on virus viability or historical specimens were also reviewed. Studies were also included if they contained experimental data on the viability of an orthopoxvirus on fomites or preserved tissue material (e.g., crusts). Studies or reports on historical specimens were included if there was suspicion of variola virus.

Existing specimens at the CDC Poxvirus Laboratory (tissues from Egypt, Italy, and England) were reexamined by using modern molecular techniques. In addition, we examined newer relic specimens (tissues from Kentucky and New York, New York, and crusts from Virginia, New Mexico, and Arkansas) for molecular signatures of poxviruses. Non-variola orthopoxvirus DNA signatures were amplified by using real-time PCR (*9*).

## Experimental Data

The infectiousness of preserved skin and tissue material from patients with smallpox has been a matter of concern, particularly as worldwide smallpox eradication was achieved (*1*,*10*–*12*). Circumstantial evidence had long placed infectious fomites as the cause of many outbreaks; however, there is little evidence that fomites were a frequent cause for disease transmission (*13*–*15*). Nevertheless, smallpox lesion material is infectious, and it is conceivable that such material was present on fomites, such as clothing, linens, and letters, and that those fomites were responsible for transmission of variola virus (*15*,*16*).

During the smallpox era, one source of live virus was lesion crusts or scabs. Crusts were successfully used for variolation in many areas before vaccination with vaccinia virus. Virus content in crusts is not correlated with the vaccination status of the patient, severity of illness, or time during the course of infection (*17*). Thus, crusts from any patient with smallpox could harbor infectious virus. Experimental studies on the infectiousness of lesion crusts, specifically in preserved specimens, are limited. However, a few experimental studies share some common conclusions about the infectious nature of crusts (Table 1).

**Table 1:** Viability of infectious variola virus in various materials*

Study, year, (reference)	Type of material	Storage conditions	Maximum storage time viable virus was recovered†
Downie and Dumbell, 1947 (*18*)	Lesion crusts	Room temperature, exposed to daylight	196 d
		Room temperature, kept in dark	417 d
		Refrigerated and then room temperature, exposed to light	>196 d after refrigeration, >341 d total)
		Refrigerated and then room temperature, kept in dark	>196 d after refrigeration, >341 d total)
		In a vacuum over calcium chloride	782 d
	Saline extract of crusts	Refrigerated	432 d
	Vesicle fluid on glass slides	Room temperature, exposed to daylight	35 d
		Room temperature, kept in the dark	84 d
	Vesicle fluid diluted in broth	Refrigerator	270 d
MacCallum and McDonald, 1957 (*19*)	Crusts embedded in raw cotton	Room temperature, indirect light	530 d
		30°C, kept in the dark, 58%, 73%, and 84% relative humidity	70, 70, and 60 d, respectively
Wolff and Croon, 1968 (*20*)	Crusts	Room temperature, kept in an envelope	4,745 d (13 y)
Huq, 1977 (*21*)	Crusts	35°C, 65%–68% relative humidity	21 d
		26°C, <10% and 85%–90% relative humidity	84 and 56 d, respectively
		4°C, 10% and 60%–62% relative humidity	112 d
		−20°C	112 d
Rao, 1972 (*15*)	Vesicle fluid on glass slides	Direct sunlight	<1 h
	Vesicle fluid in capillary tubes	Direct sunlight	<2 h

*Specimens from patients with smallpox were used for all studies. †For several studies, this is the last sampling time point and either no material was left to continue the experiment or no further samplings were conducted.

During smallpox outbreaks in the 1940s, Downie and Dumbell (*18*) tested dried crusts and vesicle fluid that were obtained from patients with smallpox (vesicle fluid was dried on glass slides before examination). Specimens were stored at room temperature and sampled at regular intervals. Viable virus was detected from vesicle fluid contained on a glass slide stored in daylight for ≤35 days and in the dark for ≤84 days. Moreover, crusts that had been stored for 417 days at room temperature and for 432 days in a refrigerator also contained viable virus. Further testing was not possible because of insufficient crust material. Nevertheless, that study was one of the first to show that viable virus could be isolated from patient material many months after collection and that optimal storage likely included dark and cool conditions (*18*).

In the mid-twentieth century, there was concern for inadvertent importation of variola virus into Great Britain in raw cotton shipped in from tropical areas (*22*). Suspicion was raised for this vehicle of importation after outbreaks occurred in British workers who handled raw cotton. An experiment was conducted to test the viability of variola virus derived from smallpox lesion crusts found in imported raw cotton (*19*). Viable virus was obtained ≤530 days from crusts stored in indirect light at room temperature. Crusts stored at higher humidity (73% and 84%) were viable until 70 and 60 days, respectively. Similar results were obtained from a study in Bangladesh, which found viable virus could be isolated from crusts stored at lower temperatures (*21*). However, crusts stored at higher temperatures and humidity did not retain viable virus after several weeks or months (*21*).

Wolff and Croon (*20*) conducted the longest study of variola viability in crusts from smallpox patients. For the study, crusts were collected from patients, individually placed in envelopes, and stored at room temperature. Viability of virus in these crusts was tested yearly for 13 years. Although the number of viable particles decreased with time, live variola virus was isolated from crusts 13 years after their initial storage. The experiment was discontinued after 13 years because crust specimens were depleted.

Further examination of variola virus viability on clothing and other objects indicated that the virus is not viable after exposure to direct sunlight for 30 min to 3 h; even indirect sunlight had an effect on viability (*15*). Although experimental studies have not yielded a well-defined period at which viable variola virus can survive in a preserved state (either deliberate experimental preservation or part of the natural process of tissue preservation), there is an overriding conjecture reached by these studies. If stored in cool, dry, and dark conditions, variola virus can survive in lesion crusts or tissues for months or years. Because each historical specimen and account is unique and the circumstances of preservation differ, it is essential to test suspicious specimens for viable variola virus.

## Historical and Scientific Accounts of Specimens

There have been several published and unpublished reports of suspected smallpox specimens surfacing since eradication (Table 2). Some reports involve scabs or crusts, and others involve entire corpses. These specimens offer an illuminating glimpse into the past, but their presence may also cause some concern for public safety in the event that any of these specimens contain viable variola virus. We present the historical and scientific accounts of each of these specimens with their respective laboratory results, which represent published and more recent data from the CDC Poxvirus Laboratory.

**Table 2:** Historical artifacts tested for variola virus and other viruses

Location, date of origination, description of the artifact (date discovered)	Laboratory testing*				
	Live virus isolated	Evidence by electron microscopy	Viral DNA isolated	Human DNA isolated	Other testing
Egypt, 1157 bce, mummy of Ramses V with lesions; lesions were present in a centrifugal distribution and had an appearance similar to smallpox (1898, 1979)	No (*2*)	No (*2*)	No†	No†	Viral particles and faint immunologic reactivity with variola antibody; negative radioimmunoassay result for smallpox (*23*)
Egypt, 1200–1100 bce, piece of skin from male mummy with a typical smallpox rash (1911)					Portion of skin did not show definite pathologic characteristics of smallpox (*24*)
Italy, sixteenth century, corpse exhumed from a crypt; lesions were umbilicated, monomorphic, and in a centrifugal distribution (1986)	No (*25*)	Yes (*25**,**26*)	No, by molecular hybridization (29); no, by DNA isolation and real-time PCR†	No†	Orthopoxvirus antigens not detected by hemagglutination or enzyme immunoassay (*25*)
Canada, 1640–1650, bones from an adult man located in a burial plot on Native American land; the tribe was known to have had a smallpox epidemic in 1634 (1966)					Bone analysis result was consistent with osteomyelitis variolosa (*27*)
Russia, late seventeenth to early eighteenth centuries, corpses exhumed from permafrost; 1 grave had multiple bodies and evidence suggested quick postmortem burial; samples were analyzed from 1 corpse (2004)			Yes, variola virus–related DNA (*28*)		
England, 1729–1856, piece of skin with lesions attached to a skeleton exhumed from a crypt (1985)	No (*29*)		No†	No†	
Russia, nineteenth century, corpses in permafrost recovered during flooding; corpses were from an area of a smallpox outbreak in the nineteenth century (1991)	No (*30*)				
Kentucky, USA, 1840–1860, mummified remains of a body with lesions discovered at a construction site (2000)	No†		No†*		
New York, New York, USA, City, mid-1800s, mummified remains of a body with lesions contained within an iron coffin discovered at a construction site (2011)	No†	No†	No†	Yes, from a tooth†	
Virginia, USA, 1876, scab from the arm of an infant to be used for community vaccination; found in letter sent from son to father in Virginia; scab was on display at a museum (2011)	No†		Yes, non-variola Orthopoxvirus DNA†	Yes†	
New Mexico, USA, late nineteenth century, scabs from vaccination sites contained in an envelope, which was contained within a book (2003)	No†		Yes, non-variola Orthopoxvirus DNA†	No†	
Arkansas, USA, 1871–1926, suspected smallpox scabs on display at a museum (2004)	No†		No†	No†	

*Published laboratory results are accompanied by the reference (number in parentheses). †Previously unpublished results.

### Corpses

An anecdote from eighteenth century England describes an outbreak of smallpox believed, at the time, to be caused by exposure to a long-buried corpse. The grave of a person with smallpox who died 30 years earlier was unearthed in the process of preparing a second grave nearby, and several of the funeral attendees became ill with smallpox (*12*,*31*). Whether these grieving attendees contracted smallpox from the graveside or from another ill person in the community, a likely occurrence during an outbreak, is unknown. However, occupationally derived smallpox infections beset mortuary workers and those who had close contact with bodies of deceased patients with smallpox. In these cases, the disease was likely contracted by contact with virus in or on the corpse or on contaminated clothing or linens (*19*,*32*). These infections may have occurred because of exposure to a recently deceased patient with smallpox, but a question remains with us now: can live virus be maintained in well-preserved ancient corpses and mummies?

### Egyptian Mummies—Twelfth Century BCE

An early examination of evidence for variola virus was conducted on a piece of skin from a male mummy housed at the Cairo Museum of Antiquities. The mummy had vesicular cutaneous lesions distributed in a pattern characteristic of smallpox. A portion of skin processed for light microscopy did not show definitive pathologic characteristics of smallpox. However, these ancient tissues were not ideally preserved for histological examination (*24*). The discovery of lesions present in a typical distribution on the mummified body of Ramses V implicated smallpox as the young pharaoh’s cause of death and shed new light on ancient Egyptian history, as well as that of variola virus (*2*). Centuries after his death, skin taken from the shroud of the mummy of Ramses V showed some viral particles and had faint immunologic reactivity (*23*); however, the sampling method was noted to have potentially been flawed and no live virus or viral DNA was isolated or amplified from specimens (*2*). Human DNA was also not detected in these specimens. Thus, although there is no laboratory data to firmly support a postmortem diagnosis, the visual appearance was suggestive of a variola infection before his death (*2*).

### Archeologic Excavations

There have been 2 examples of corpses exhumed from crypts during archeologic excavations in the twentieth century. In both examples, the corpses had what were described as typical variola lesions, and the bodies had been contained in cool, dark environments. No live virus, viral DNA, or human DNA remained within these corpses. However, a corpse from sixteenth century Italy showed immunologic electron microscopy results that were consistent with those expected for orthopoxvirus infection (*25**,**26**,**29*). An archeologic excavation of a known Native American grave site (1640–1650) in Ontario, Canada, recovered bones from an adult male. The bones had visual scarring and an appearance consistent with osteomyelitis variolosa, a disease manifestation of smallpox in the bones and joints. On the basis of extensive document review and bone analysis, the investigators determined that the person likely had smallpox before 1639 and survived the infection with long-term osteomyelitis variolosa (*27*).

### Permafrost in Russia

Two corpses with questionable lesions and that had been contained within permafrost in Siberia have been unearthed: one was unearthed naturally during flooding, and the other during an archeologic excavation. Dating of the corpses to the late seventeenth or early eighteenth century matched with written accounts of smallpox epidemics in the local communities for one of the sites, but no live virus was obtained from these remains (*28**,**30*). The more recent archeologically excavated corpse was sampled as soon as graves and mummified remains were exposed to the surface. The corpse yielded DNA closely related to more recent variola virus specimens. This finding provided further insight into the strain of variola that was circulating in northeastern Siberia during the late seventeenth or early eighteenth centuries (*28*).

### Construction Sites in Kentucky and New York, New York, USA—Nineteenth Century

**Figure 2:**
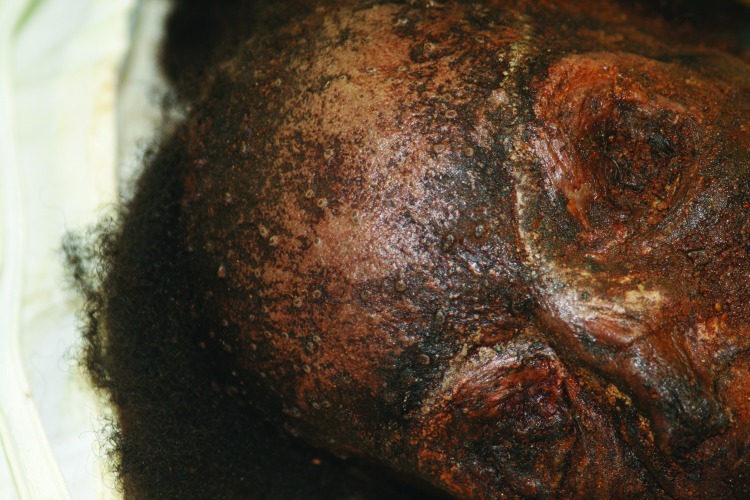
Mummified remains of a woman buried in an iron coffin, New York, New York, USA, mid-1800s. Photograph provided by Don Weiss.

There are 2 accounts of remains with suspicious lesions that were accidently unearthed during construction at a burial site. In 2000, mummified remains were discovered at a construction site in Kentucky. No live virus or viral DNA was isolated from these remains. More recently in 2011, the remains of a woman buried in an iron coffin were uncovered during construction at a known African-American cemetery in New York, New York. Preservation of the body was remarkable because of the airtight environment provided by the iron coffin (*33*). The presence on the body of lesions with the characteristic deep-seated, umbilicated appearance and in a centrifugal distribution of smallpox lesions immediately prompted concern for unearthed smallpox (Figure 2). No live virus or viral DNA was isolated from or visualized in any of multiple specimens taken from the body and evaluated by cell culture, molecular methods, or immunohistochemical stains. Human DNA was isolated from a tooth pulp specimen. Thus, the results do not conclusively verify the hypothesis of smallpox as the cause of death. However, visual inspection cast little doubt on this hypothesis.

### Crusts from Patients

Some accounts from the eighteenth century report that material used in variolation (often scab material) was stored for ≤8 years before successful use (*34*). Thus, long-term storage and subsequent use of variola virus from preserved specimens have long been recognized. However, during the era of eradication, 45 scab specimens were collected from variolators and tested 9 months after collection; live virus was not isolated from any of the specimens (*35*). Nevertheless, stored crusts have caused immediate concern for potential exposures and their discovery has caused immediate exposure mitigation and testing.

**Figure 3:**
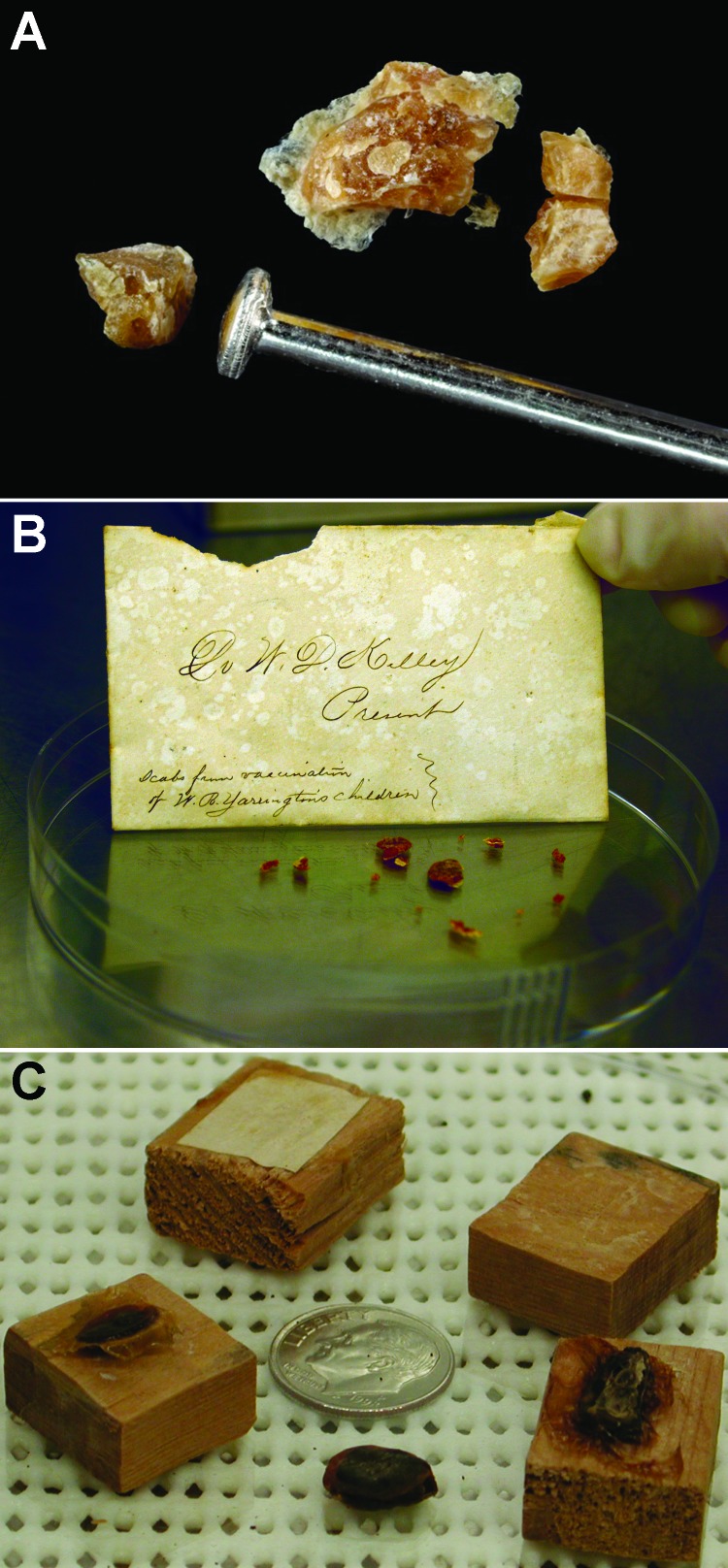
Recovered crusts. A) Lesion crust material from Virginia, USA, photographed after gamma irradiation. Photograph by James Gathany. B) Lesion crust material from an envelope contained within a book, New Mexico, USA, nineteenth century. Photograph by Russell L. Regnery. C) Lesion crust material from a jar on display in a museum, Arkansas, USA. Photograph provided by Erin Goldman.

In the past 10 years, suspected variola crusts have been discovered in the United States on 3 occasions. In Virginia, a crust labeled as a smallpox scab was on display at a museum and was accompanied by a letter describing its origin (Figure 3, panel A). The letter and crust were sent from 1 family member to another in Virginia in 1876, and the correspondence stated that the crusts came from the arm of an infant and were to be used to vaccinate others. No live virus was isolated from this crust. However, non-variola orthopoxvirus DNA and human DNA were successfully extracted. This rare letter and scab are evidence to support arm-to-arm vaccination in the United States around the same time that it was also performed in Great Britain (*7*).

A second incident of suspected smallpox scabs on display at a local museum occurred in Arkansas (Figure 3, panel B). These relics were donated by the family of a physician who practiced in Arkansas during 1871–1926. In 1905, there was a large smallpox outbreak in Arkansas (*36*). No live variola virus, viral DNA, or human DNA were isolated from the specimens. The crusts were affixed to blocks of wood with a dense resin, and the resin may have been inhibitory to the PCR or DNA stability. The origins and species of these specimens will continue to remain a mystery.

In 2003, a librarian in New Mexico opened a book and an envelope containing lesion crusts fell out of the book (Figure 3, panel C). The envelope was labeled “scabs from vaccination of W.B. Yarrington’s children,” and the book was dated 1888. Similar to the relic from Virginia, no live virus was isolated from this material, but non-variola orthopoxvirus DNA was isolated. In this instance, human DNA was not amplified. The question of precisely what virus was used in vaccination in the United States in the nineteenth century is intriguing from the perspective of historical significance and the evolution of orthopoxviruses.

## Public Health

Historical specimens come to the attention of public health authorities when there is a perception that they may constitute a potential risk to those who are handling or may have handled the artifacts. This concern extends to specific groups of persons who might work routinely with historical specimens, including archeologists and museum archivists, as well as those who may stumble upon these specimens on an irregular basis, such as construction workers or the general public. Although live variola virus has never been isolated from historical tissues, this finding does not eliminate the possibility of live variola virus resurfacing from well-preserved tissue material (*10*,*12*). Moreover, variola virus has been absent for >30 years, and there is an increasingly large population of susceptible persons who have never been vaccinated against smallpox.

The discovery of a series of corpses and mummies with suspected smallpox lesions in the late 1970s and 1980s sparked a series of commentaries over the risks to archeologists and anthropologists and the potential need for vaccination of workers (*19**,**23**,**33**,**34*). This proposition has been hotly debated, and opponents have argued that live variola virus has never been isolated from archeologic specimens and that live virus vaccination carries its own risks. This debate underscores the lack of firm scientific evidence to enable an informed assessment of risk to those who come into contact with artifacts and relics potentially contaminated with variola virus. The inability to exclude the possibility of risk led to the vaccination of 3 archeologists who handled a corpse with suspect lesions in London in 1985 (*29*). Current recommendations from the Advisory Committee on Immunization Practices do not specifically address vaccination for those who work with antiquities, including corpses and tissue material (*6*). Although routine vaccination is not recommended, prudent preparation and recognition of potential smallpox relics is advised for those who work with potentially contaminated tissues and corpses (*29*).

If a suspected smallpox relic or body of a person who died of smallpox has been discovered, local and state public health departments are an excellent resource. Public health officials can work closely with those who have handled any suspect artifacts, determine risks, help mitigate concern, and arrange for appropriate testing. Testing can be performed on a suspected specimen to definitively determine if live virus is present. The WHO smallpox reference laboratories can perform these tests and have successfully participated in inquiries involving historical specimens (Table 1).

## Conclusions

Aside from immediate public health concerns surrounding a suspected smallpox specimen, historical cases help highlight disease history in terms of the society and patient in question. Historical specimens might also help explain the history of smallpox epidemics and vaccine development. Recent exhumation of a corpse from permafrost in Siberia led to sequence characterization of an older strain of variola virus, which shed light on the evolutionary history of the virus (*28*).

Today, the smallpox vaccine consists of an intradermal inoculation with vaccinia virus, and the premise and method of this vaccination has not changed since the time of Jenner (*7*). However, the species of virus that Jenner used to vaccinate persons is still debated (*37*). Irrespective of the debate, most scientists agree that Jenner and generations of persons since him have used an orthopoxvirus species in vaccinations to confer immunity to smallpox. Accounts of vaccination exist in historical records, but descriptions of which virus was used, how it was used, and who was performing procedures (e.g., physicians, communities) are sparse. Thus, our understanding of the history of smallpox vaccination is incomplete. Information obtained from historic relics helps build an understanding and picture of vaccination before the twentieth century (*1*).

Modern molecular approaches can be used with historical specimens to confirm the presence of variola or another orthopoxvirus and elucidate the evolutionary history of the virus. Full genome, gene, or partial gene sequencing of isolates enables investigating the history of 1 virus compared with others. Long-term stability of smallpox virus DNA is not well characterized. However, constant low temperatures, such as those in crypts and permafrost, are believed to be key to the stability of DNA molecules. Theoretically, DNA can survive up to ≈1 million years in cold environments (*38*). Specific characteristics that make orthopoxviruses stable and viable over long periods are unknown. However, for viruses embedded in tissue (such as those in crusts or skin specimens), it is reasonable to postulate that being surrounded by a protein or organic matrix may provide some protection to the virus.

Archival specimens offer opportunities to delve into the past and capture a glimpse of the history of an eradicated disease. There are no published reports of residual live microbes found in archeologic relics. Furthermore, on the basis of experiences in the past several decades, risks for transmission of live organisms from such relics would seem to be nonexistent; nevertheless, archeologic specimens should be handled with caution. Each situation should be approached independently and with vigilance and attention. Special attention to the scientific value of a specimen will yield useful data about smallpox and vaccination history that might provide useful information about the virus and affected populations.
